# Elaboration of bilateral symmetry across *Knautia macedonica* capitula related to changes in ventral petal expression of *CYCLOIDEA*-like genes

**DOI:** 10.1186/s13227-016-0045-7

**Published:** 2016-03-31

**Authors:** Brent A. Berger, Veronica Thompson, Aedric Lim, Vincent Ricigliano, Dianella G. Howarth

**Affiliations:** Department of Botany, University of Wisconsin-Madison, 430 Lincoln Dr., Madison, WI 53706 USA; Department of Biological Sciences, St. John’s University, 8000 Utopia Parkway, Queens, NY 11439 USA

**Keywords:** *CYCLOIDEA*, Floral symmetry, *Knautia*, Capitula, qPCR

## Abstract

**Background:**

Shifts in floral form across angiosperms, particularly from radially symmetrical to bilaterally symmetrical flowers, are often associated with shifts in speciation rates and changes in pollination syndrome. Growing evidence across both rosids and asterids indicates that *CYCLOIDEA* (*CYC*)-like transcription factors from the TCP gene family play a role in establishing the dorsoventral pattern of flower symmetry, which affects the development of both the corolla and androecium. Previous studies of *CYC*-like genes, especially of the CYC2 clade, indicate that these genes are dorsally restricted in bilaterally symmetrical flowers. Also, gene duplication of *CYC*-like genes often correlates with shifts in floral form in both individual flowers and head-like inflorescences (capitula).

**Results:**

Here, we compared the expression patterns of six *CYC*-like genes from dorsal, lateral, and ventral petals of internal and external florets across capitula of *Knautia macedonica* (Dipsacaceae). We demonstrate that multiple copies of *CYC*-like genes are differentially expressed among petal types and between internal and external florets. Across paralogs, there was a general trend toward a reduction in dorsal expression and an increase in ventral expression in internal florets compared to external florets. However, it was in the ventral petals where a statistically significant increase in expression correlates with a less zygomorphic flower. We also show for the first time lateral-specific expression of a *CYC*-like gene. Additionally, dorsoventral asymmetric expression of a CYC3 paralog indicates that this understudied gene clade is likely also involved in floral symmetry.

**Conclusions:**

These data indicate that the elaboration of bilateral symmetry may be regulated by the dorsoventral gradient of expression, with statistically significant changes in ventral expression correlating with changes in dorsoventral morphological specialization.

**Electronic supplementary material:**

The online version of this article (doi:10.1186/s13227-016-0045-7) contains supplementary material, which is available to authorized users.

## Background

Shifts in dorsoventral asymmetric expression of transcription factors affecting growth and patterning of the corolla and androecium of a flower can give rise to a vast array of different floral symmetries that potentially affect reproductive strategies and plant evolution. Across angiosperms, major transitions in floral symmetry between radially symmetrical (actinomorphic) and bilaterally symmetrical (zygomorphic) flowers have been common [1–3]. Such morphological changes are of special interest in relation to pollination (e.g., see [4]) and perhaps to rates of speciation [5]. Additionally, bilaterally symmetrical flowers vary significantly across groups in the degree of asymmetry along the dorsoventral axis. A limited number of clades vary in floral symmetry across a single inflorescence, with multiple types of florets with different degrees or types of symmetry occurring. Bilaterally symmetrical flowers generally have three different morphological modules: dorsal petals, lateral petals, and a single ventral petal [6], suggesting that genes regulate the development of these petals differently.

Numerous studies across angiosperms show that morphological shifts between radially symmetrical and bilaterally symmetrical flowers are correlated with independent transitions in asymmetric dorsoventral expression of members of the ECE clade of TCP transcription factors [7–9]. *CYCLOIDEA* (*CYC*) was the first characterized member [10] and has been shown repeatedly to play a role in specifying dorsal petal identity [9–12]. Around the divergence of the core eudicots, *CYC*-like genes duplicated twice, resulting in three copies—CYC1, CYC2, and CYC3 [7]. Within the core eudicots, studies of the role of *CYC*-like genes in floral symmetry have focused on the CYC2 clade of core eudicots [7]. The dorsal restriction of CYC2 genes has been found in all bilaterally symmetrical core eudicots to date [12, 13]. In contrast, in radially symmetrical groups, CYC2 clade members either are not expressed in corolla tissue or are ubiquitously expressed [14, 15]. The localization of CYC2 has been determined with in situ hybridization and/or semiquantitative RT-PCR from dissected petal lobes (see [9, 12]). Additionally, it has been shown across core eudicots that loss of expression of CYC2 paralogs results in ventralization of the flower [10, 11, 16–18]. All of these data suggest that CYC2 genes are dorsal identity genes.

In Dipsacales, the first transition from radial to bilateral symmetry is correlated with a duplication event, resulting in two CYC2 and two CYC3 gene copies [19]. Previous work indicates that the expression of both CYC2 copies is partially restricted to the dorsal and lateral corolla lobes [14]. Subsequent shifts to more strongly bilaterally symmetrical flowers involved further restriction of the duplicate copies and a decoupling of expression, such that one copy is more dorsally restricted than the other [14]—a pattern observed in other core eudicots (*Antirrhinum majus* [11]; Malphigiaceae [15]; and *Pisum sativum* [16]). This variation in paralog expression strongly suggests that differentially restricted expression of CYC2 copies occurs independently in the evolution of bilateral symmetry from radially symmetric ancestors.

Additional independent duplications of *CYC*-like genes have been common within bilaterally symmetrical groups, especially in the CYC2 clade (e.g., [9, 20]). With the exception of six genera in the Brassicaceae whose symmetry is regulated by a single CYC2 ortholog [21, 22], all other examined bilaterally symmetrical core eudicots have two or more paralogs belonging to the CYC2 clade. The other two core eudicot clades of ECE *CYC*-like genes (CYC1 and CYC3; see [7]) have not been well examined, but independent duplications have been found in Caprifoliaceae (including Dipsacaceae) and Asteraceae [17–19, 23]. In Caprifoliaceae, a duplication in CYC3 is correlated with the transition to bilateral symmetry, suggesting that these paralogs may also play a role in determining floral symmetry.

Plants with capitula (compact floral head inflorescences) comprised of both radially and/or multiple types of bilaterally symmetrical flowers, such as those in Asteraceae and Dipsacaceae, appear to have the greatest number of CYC2 paralogs [23–25] with differential expression tied to changes in floral symmetry across the inflorescence axis [18]. Although not as commonly examined, Asteraceae and Dipsacaceae also have duplicated CYC1 and CYC3 clades [18, 23, 24]. A typical capitulum consists of many small flowers (florets) packed tightly into a condensed head that can closely resemble a single, large flower. In some groups (e.g., Asteraceae, *Actinodium* of Myrtaceae) capitula have small, similar florets throughout the inflorescence with the exception of the outer ring of much larger, more strongly bilaterally symmetrical flowers [26]. In others (e.g., Dipsacaceae), florets gradually increase in size from the center (internal florets) to the outer ring (external florets) [24].

**Fig. 1:**
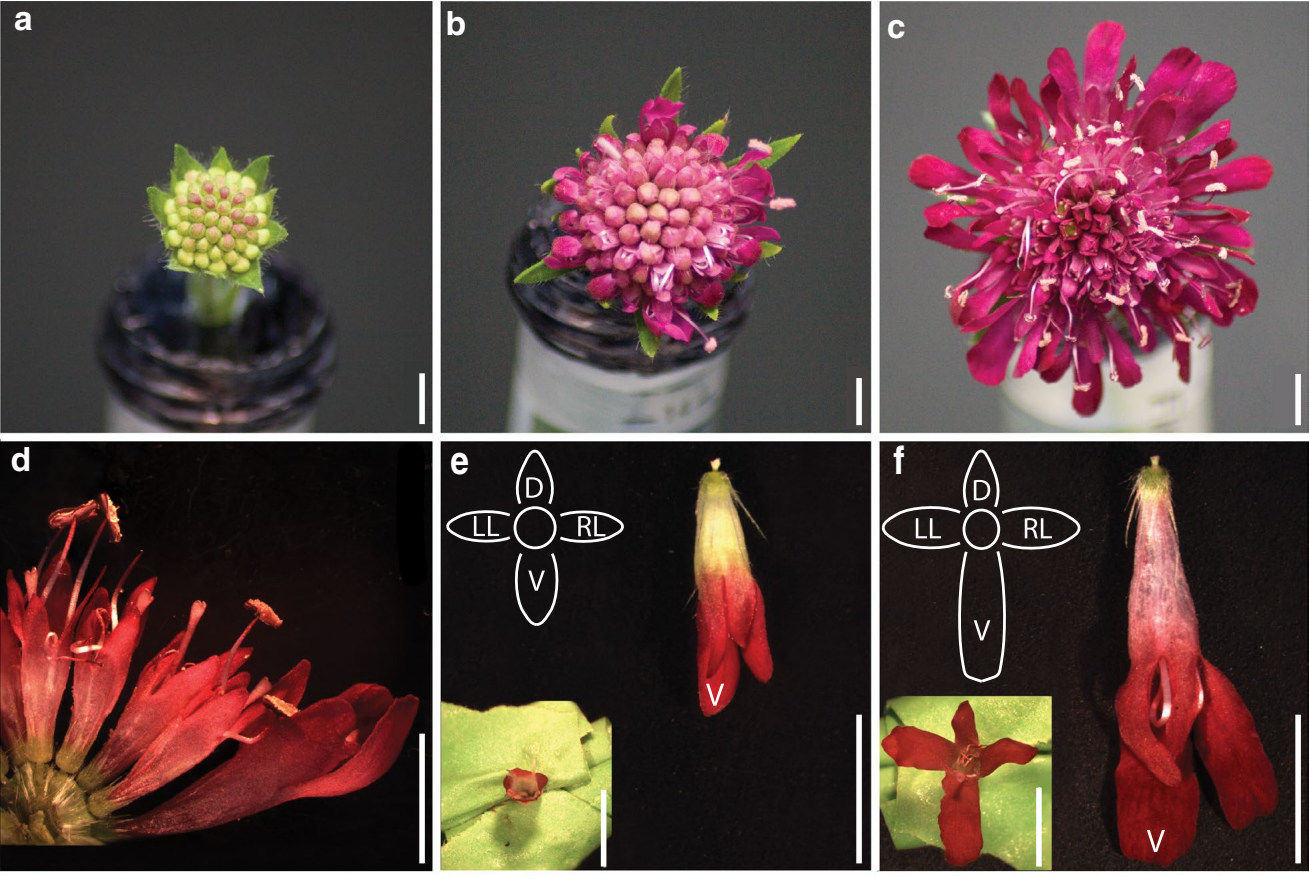
Floral images of *Knautia macedonica* capitulum development. **a**–**c** Developmental series of capitulum development. **d** Cross section through a mature capitulum. **e**, **f** Representative examples of internal and external florets. *Diagrams* show orientation of petals in **e**, **f** with *D* dorsal, *LL* left lateral, *RL* right lateral, and *V* ventral. (*Scale bars* 5 mm)

*Knautia macedonica* (Dipsacaceae) has four-parted weakly zygomorphic internal florets that gradually expand and diverge across the dorsoventral axis into strongly zygomorphic florets in the external ring (Fig. 1). This variation in the differentiation of dorsoventral specialization across the inflorescence of *K. macedonica* provides an opportunity to quantify gene expression of *CYC*-like paralogs among natural variation in bilateral symmetry. Previous studies examining the *CYC*-like gene phylogeny and duplication events in Dipsacaceae indicated that this family had perhaps the greatest number of paralogs of any examined group with as many as 17 paralogs across the ECE clade (CYC1 = 5, CYC2 = 9, CYC3 = 3) [24]. Not all paralogs were found in all species; however, at least a few could have been the result of allelic variation. Additionally, frequent changes in ploidy number, even within species [27], could be complicating simple identification of gene number.

The aim of the present study was to quantify ECE *CYC*-like paralogs from petal dissections of internal and external florets from *K. macedonica*. We used qPCR with ANOVA and post hoc Tukey HSD statistical tests to examine significant changes in expression of 6 *CYC*-like paralogs across the dorsoventral axis. Our important findings include that: (1) although weakly expressed, the expression of 4 *CYC*-like paralogs is significantly increased in the ventral petals of internal florets; (2) a CYC2 paralog is restricted specifically to lateral petals; and (3) a CYC3 paralog likely also plays a role in specifying dorsoventral symmetry.

## Results

### Phylogenetic analyses

The aligned matrix of 165 *CYC*-like genes included 546 nucleotides spanning the 3′-end of the TCP domain through the 5′-end of the R-domain. The dataset is deposited in TreeBASE (http://purl.org/phylo/treebase/phylows/study/TB2:S18401). The six *K. macedonicaCYC*-like genes fall into 3 main clades representing CYC1, CYC2, and CYC3 based on maximum likelihood (ML) and Bayesian inference (BI) analyses (see Additional file 1). These three clades, as well as subclades CYC2A, CYC2B, CYC3A, and CYC3B, are consistent with previous findings examining *CYC*-like genes across eudicots [7, 9, 14, 19, 23, 24, 28]. With the exception of subclade CYC3A, all clades are supported by ML bootstrap values (BS ≥ 86) and BI posterior probabilities (PP ≥ 0.99). Subclade CYC3A is well supported by PP ≥ 0.99; however, ML BS < 70. Four of the six *K. macedonicaCYC*-like genes are sister to their corresponding paralog of *Knautia calycina* with moderate to high support (*KmCYC1* BS = 99, PP = 1.0; *KmCYC2A* BS = 74, PP = 0.70; *KmCYC2Ba* BS = 86, PP = 1.0; *KmCYC3B* BS = 100, PP = 1.0). The *KmCYC2Bb* gene is part of an unresolved basal grade of CYC2Bb copies that also include CYC2Bb from *Knautia calycina*. The remaining *KmCYC3A* gene from *K. macedonica* has no corresponding *K. calycina* paralog but is placed in a well-supported clade (BS = 99, PP = 1.0) with other Dipsacaceae CYC3A genes. Based on our analyses (Additional file 1), gene duplication and subsequent coalescence have been integral processes in the evolution of the Caprifoliaceae (including Dipsacaceae). The topology and the location of *CYC*-like gene duplication events are largely in agreement with prior studies [14, 19, 24].

### qPCR expression analyses

**Fig. 2:**
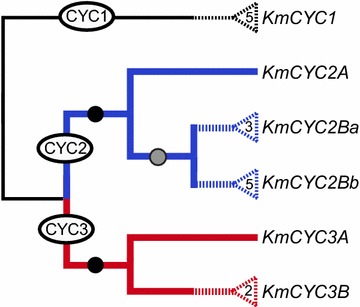
Putative gene tree of *CYC*-like paralogs in *Knautia macedonica*. Duplication events that span the core eudicots are shown in *black* (CYC1), *blue* (CYC2), and *red* (CYC3). *Black circles* label duplications that span the Caprifoliaceae. *Gray circles* indicate a putative Dipsacaceae duplication. A single paralog was successfully cloned and used in qPCR experiments from each major clade. Additional paralogs are hypothesized within other groups of Dipsacaceae and are shown as *dashed lines* with putative copy numbers indicated inside *triangles*

**Fig. 3:**
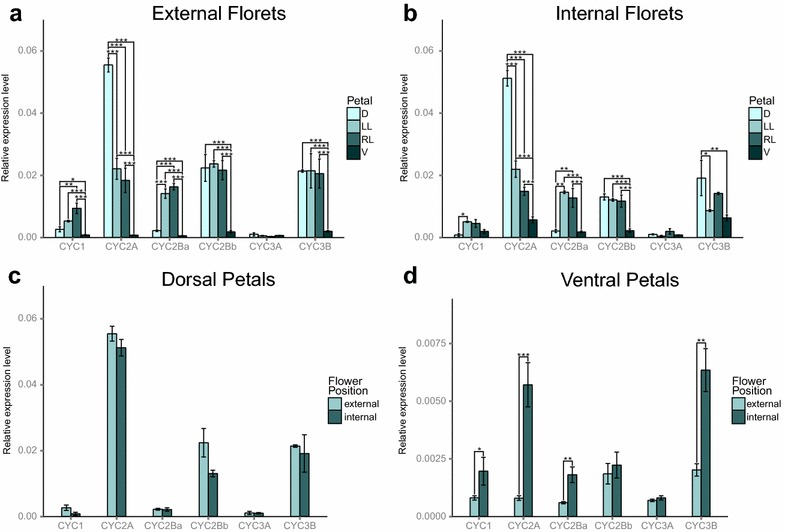
qPCR relative expression of *CYC*-like paralogs for external and internal florets. **a** Relative expression pattern for external petal types by paralog. **b** Relative expression pattern for internal petal types by paralog. **c** Relative expression pattern for dorsal petals. **d** Relative expression pattern for ventral petals (note difference in y axis values). Abbreviations for petal types are as follows: *D* dorsal petals, *LL* left lateral petals, *RL* right lateral petals, and *V* ventral petals. *Standard error bars* are shown. Statistical significance among petal types is as follows: *p ≤ 0.05; **p ≤ 0.01; ***p ≤ 0.001

RNA extracted from leaves, internal buds, and external buds of *K. macedonica* indicate differential expression of all *CYC*-like genes between the two tissue types (i.e., leaf and floral bud), as well as between internal and external buds (Additional file 2). Relative transcript abundance was significantly higher in floral buds than leaves for *KmCYC2A* (*p* ≤ 0.01), *KmCYC2Ba* (*p* ≤ 0.01), and *KmCYC2Bb* (*p* ≤ 0.01) and weakly significant comparing external buds to leaves in *KmCYC3B*. *KmCYC1* and *KmCYC3A*, on the other hand, were significantly higher in leaves than at least one floret type. There was no statistically significant difference between external and internal buds in any gene. Additionally, there was no significant difference between left lateral and right lateral expression in any *CYC*-like paralog (Figs. 2, 3).

Relative transcript abundance of *CYC*-like genes varied among petal types for both internal and external florets (Fig. 3; Additional file 3). Although similar in overall expression pattern to internal florets (Fig. 3a), external floret transcript abundance had more significant differences among petal types than was detected in internal florets. Dorsal (*p* ≤ 0.05), left lateral (*p* ≤ 0.01), and right lateral petals (*p* ≤ 0.001) were all significantly higher than ventral petals for *KmCYC1*. Dorsal petals were significantly higher for *KmCYC2A* than left lateral (*p* ≤ 0.001), right lateral (*p* ≤ 0.001), and ventral petals (*p* ≤ 0.001). Both lateral petals were also significantly higher in transcript abundance for *KmCYC2A* compared to ventral petals (*p* ≤ 0.001). *KmCYC2Ba* expression in external florets was significantly higher in left and right lateral petals compared to both dorsal and ventral petals (*p* ≤ 0.001). Although very weak, dorsal petal expression was also significantly higher than ventral expression in external florets (*p* ≤ 0.001). In external florets, dorsal, left lateral, and right lateral petals all had significantly higher *KmCYC2Bb* expression than ventral petals (*p* ≤ 0.001). The expression levels of *KmCYC3B* in dorsal (*p* ≤ 0.001), left lateral (*p* ≤ 0.001), and right lateral (*p* ≤ 0.001) petals were all significantly higher than ventral petals. No differences in relative expression levels were detected among petal types for *KmCYC3A*, which had barely detectable levels of expression overall.

Regarding internal florets (Fig. 3b), dorsal petals had a significantly lower level of *KmCYC1* transcript abundance compared to left lateral petals (*p* ≤ 0.05). *KmCYC2A* was significantly higher in dorsal petals compared to left lateral (*p* ≤ 0.001), right lateral petals (*p* ≤ 0.001), and ventral petals (*p* ≤ 0.001). Both left and right lateral petals also had a significantly higher level of *KmCYC2A* transcript abundance compared to ventral petals (*p* ≤ 0.001). Similar to external florets, the expression of *KmCYC2Ba* was predominately in lateral petals and was significantly higher than dorsal (*p* ≤ 0.01) and ventral petals (*p* ≤ 0.001). Unlike external florets, there was no significant difference between dorsal and ventral expression of *KmCYC2Ba*. Significantly higher relative expression levels (*p* ≤ 0.001) were detected for *KmCYC2Bb* when comparing dorsal, left lateral, and right lateral petals to ventral petals. No significant difference in expression of *KmCYC3A* was seen among any petals. *KmCYC3B* was more highly expressed in dorsal petals than left lateral (*p* ≤ 0.05) or ventral (*p* ≤ 0.01).

Dorsal petals showed no significant change in expression between external and internal florets for any paralog (Fig. 3c), although four paralogs (*KmCYC1*, *KmCYC2A*, *KmCYC2Bb*, and *KmCYC3B*) showed a reduction in expression in internal florets. Ventral petals of internal florets, however, did show a significant increase in expression for *KmCYC1*, *KmCYC2A*, *KmCYC2Ba*, and *KmCYC3B* (Fig. 3d). Across paralogs, there was a general trend toward a reduction in dorsal expression and an increase in ventral expression in internal florets compared to external florets.

## Discussion

### Reduction in dorsoventral disparity of gene expression and morphology correlated

Across diverse core eudicots, it has been shown that the transition from radial to bilateral floral symmetry is correlated with dorsal restriction of CYC2 genes [9, 14, 15]. Our previous work in Caprifoliaceae with semiquantitative RT-PCR suggested that ubiquitous expression of CYC2 genes in radially symmetrical groups became more dorsally restricted in successively nested clades of increasing dorsoventral specialization [14]. Here, we quantify the expression of six *CYC*-like paralogs in *K.macedonica,* which suggests that it is not simply loss of expression in certain petal lobes that governs symmetry, but the disparity in expression across the dorsoventral axis. Most studies have examined *CYC* expression using in situ hybridization (see [9]), which does not pick up weak ventral expression and therefore would not uncover this pattern.

**Fig. 4:**
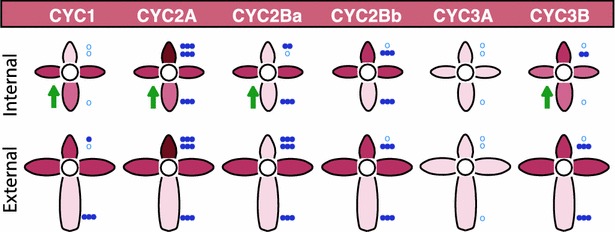
*Knautia macedonica* expression model for internal and external florets. *Color shade* indicates level of expression with *darker shades* signifying higher expression relative to other petals in that flower. Significance shown in *blue*: *circles* (not significant) and *blue asterisk* (*p ≤ 0.05; **p ≤ 0.01; ***p ≤ 0.001). *Blue circles* or *asterisks* show dorsal to lateral (*top*), dorsal to ventral (*middle*), and lateral to ventral (*bottom*). *Green arrows* highlight petals with a significant change in expression between external and internal florets

In radially symmetrical flowers, CYC2 genes appear to be uniform across the dorsoventral axis, either expressed across the axis, or not expressed at all [14, 15, 29–31]. The only empirical studies of this expression pattern in radially symmetrical flowers have been in Malpighiales [15], where the data suggest that expression is uniform across dorsal, lateral, and ventral lobes, and in cruciferous Brassicaceae, where a similar pattern of uniform expression is observed between adaxial and abaxial pairs of petals in mature flowers [22]. Data from bilaterally symmetrical groups using in situ hybridization or semiquantitative RT-PCR indicate that CYC2 paralogs may be absent from ventral lobes and restricted dorsally, or they may be asymmetrically expressed as in bilaterally symmetrical Brassicaceae [21, 22]. Our qPCR data indicate that even though expression in ventral petals of *K. macedonica* is very weak, the variation in the elaboration of symmetry correlates with significant differences not in the dorsal petals, but in the ventral petals. Specifically, four paralogs (*KmCYC1*, *KmCYC2A*, *KmCYC2Ba*, and *KmCYC3B*) are all upregulated in internal florets compared to external florets (Fig. 4).

**Fig. 5:**
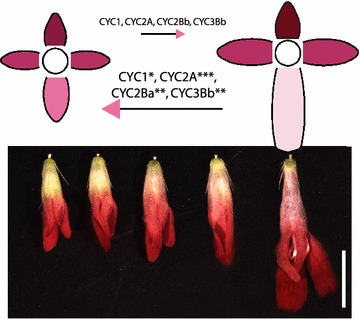
Model of *CYC*-like paralog expression across the inflorescence. In the transition from internal to external florets, there is a slight reduction in expression of four *CYC*-like paralogs in the dorsal petals. Conversely, there is a slight increase in all six paralogs (three of which are significant) in ventral petals. This results in an evening out of asymmetry along the dorsoventral axis in internal florets. *Asterisk* denotes level of significance: *p ≤ 0.05; **p ≤ 0.01; ***p ≤ 0.001

Although most of the expression differences between internal and external florets are not significantly different, there are general trends that indicate a difference in disparity along the dorsoventral axis. Comparing the dorsal petals of internal florets to external florets, the mean expression of *KmCYC1*, *KmCYC2A*, *KmCYC2Bb*, and *KmCYC3B* is reduced. In contrast, in ventral petals the mean expression of all six paralogs is increased, four of which are statistically significant. Collectively, these data suggest that across multiple paralogs from CYC1, CYC2, and CYC3, there is a trend that dorsoventral gene expression forms a gradient that becomes more pronounced with increasing dorsoventral morphological specialization (Fig. 5).

### Subtle changes across multiple paralogs correlate with change in degree of zygomorphy

Our findings support the hypothesis that the degree of zygomorphy exhibited by flowers in capitula of *K. macedonica* is influenced by subtle changes in the expression levels of multiple *CYC*-like genes (hypothesized in [14]). Specifically, we have shown that the six paralogs we were able to successfully clone and examine in this study from the CYC1, CYC2, and CYC3 clades are differentially expressed among dorsal, lateral, and ventral petals, as well as between internal and external florets. Additionally, we show that the pattern of differential expression between internal and external florets differs depending on the *CYC*-like paralog. Given all available evidence, we hypothesize that capitula in *K. macedonica*, and likely other closely related taxa with similar inflorescences [24], demonstrate subtle shifts in expression levels of multiple *CYC*-like paralogs that in turn influence the amount of differentiation along the dorsoventral axis of internal and external florets. We successfully cloned six ECE *CYC*-like paralogs in *K*. *macedonica* with at least one from each of the three major clades in Dipsacaceae (i.e., CYC1, CYC2, and CYC3) [24]. Prior work found evidence of additional duplications within each of these clades [24] and suggested Dipsacaceae underwent dynamic changes in chromosome number in many species; however, recent studies demonstrated that *Knautia macedonica* is a diploid species and has a relatively small genome size [27, 32, 33].

A few studies have examined dimorphism between internal and external florets and its relation to *CYC*-like gene expression in the sunflower family (Asteraceae) where clear delineation or transitional morphs are observed between radially symmetrical disk florets and bilaterally symmetrical ray florets (e.g., *Helianthus annuus* [17, 23, 34, 35], *Gerbera hybrida* [18], and *Senecio vulgaris* [36]). Developmental differences in Asteraceae floret morphology are often attributed to differential expression of *CYC*-like paralogs, resulting from ancient paleopolyploidization events that occurred after the evolution of the core eudicots and around the divergence of Asteraceae [7, 23, 37]. These differential patterns were examined by comparing the presence of *CYC*-like gene expression in disk versus ray florets and have shown that there are paralogs specific to each floret type [25].

In a similar fashion, Dipsacaceae has undergone multiple, independent whole-genome duplications, resulting in the retention of numerous *CYC*-like paralogs [19, 24], that, as we demonstrate here, have also diverged in expression pattern (i.e., paralogs are expressed at different levels in different petal types; Figs. 3, 4). The divergent expression pattern of paralogs belonging to the same *CYC*-like clade (e.g., CYC2A and CYC2B) in internal and external florets of *K.macedonica* has been documented in other plant lineages lacking capitula and possessing bilaterally symmetrical flowers (e.g., Malpighiaceae, [15, 31, 38]; Lamiales [28]; Fabales [16]; and other clades in Dipsacales [14]). *KmCYC2A*, much like its ortholog *DipsCYC2A* in *Diervilla sessilifolia* and *Lonicera sempervirens* [14], has the highest expression of any *CYC*-like gene and is expressed predominantly in dorsal petals with significantly lower expression in lateral petals and very weak expression in ventral petals in both florets. In a similar fashion, one paralog of CYC2B, *KmCYC2Bb*, has the highest expression in dorsal and lateral petals with little expression in ventral petals—a pattern also observed for *DipsCYC2B* in *Diervilla sessilifolia*, *Lonicera morrowii*, *L*. × *bella*, and *L. pericylmenum* [14].

### A lateral-specific CYC2B paralog

While a single CYC2B gene has been detected in most Dipsacales lineages [19], prior data suggest Dipsacaceae possess additional paralogs with at least two CYC2B copies in *Knautia macedonica* (*KmCYC2Ba* and *KmCYC2Bb*; this study). As previously mentioned, *KmCYC2Bb* is expressed throughout dorsal and lateral petals in a similar pattern as its ortholog *DipsCYC2B*. The second paralog, *KmCYC2Ba*, is primarily expressed in lateral petals with little expression in either the dorsal or ventral petals, which to our knowledge represents the first documented instance of a lateral-specific *CYC*-like gene expression pattern. This pattern is seen in both external and internal florets. Initial studies of *CYC* function in *Antirrhinum* showed changes in the number of dorsal petal organs [11], so it is possible that the loss of expression of this paralog in the dorsal region is related to the shift to a single dorsal petal. Of note, despite very low expression in external florets, *KmCYC2Ba* expression is higher in dorsal petals than ventral ones, while there is no significant difference among these petals in internal florets. This is likely attributed to a significant increase in ventral petal expression in internal florets (Fig. 3d).

### Importance of CYC1 and CYC3 paralogs

While great attention has been dedicated to CYC2 genes because of their role in establishing dorsoventral asymmetry in flowers, little is known about the expression patterns of CYC1 and CYC3 genes in bilaterally symmetrical flowers, or the role they may play in inflorescence development. Prior studies of the *CYC1*-like orthologs *BRANCHED1* (*AtBRC1*) in *Arabidopsis* [39, 40], *OsTB1* in rice [41], *PsBRC1* in pea [42, 43], and two *BRC1* paralogs in tomato [44], as well as qPCR analysis of two *CYC1*-like paralogs in the pseudanthium of *Actinodium* (Myrtaceae) [26], all support a role in arresting axillary bud growth and, thus, influence overall branching pattern. *KmCYC1* is most highly expressed in leaves and is only weakly expressed in flower buds (Additional file 2). Although weakly expressed in flower buds, there are significant differences between dorsal, lateral, and ventral petals in external florets. Additionally, as with of CYC2 and CYC3, there is a significant increase in expression in ventral petals in internal florets. These data suggest that *KmCYC1* may play some role in determining symmetry.

It was previously suggested that a CYC3 gene in *Helianthus* may also be part of the floral symmetry pathway, given that it was more highly expressed in bilaterally symmetrical ray florets than in radially symmetrical disk florets [25]. However, the expression of *KmCYC3B* in *Knautia* provides strong evidence, for the first time that CYC3 paralogs are asymmetrically expressed across the dorsoventral axis. In external florets, which are strongly zygomorphic, *KmCYC3B* has a similar expression pattern to *KmCYC2Bb*, in which dorsal and lateral petal expression is significantly higher than ventral expression. Therefore, *KmCYC3B* has asymmetric expression across the dorsoventral axis. In internal florets, which are weakly zygomorphic, the asymmetric expression is only strongly significant between dorsal and ventral petals. Lateral petal expression is not significantly different than ventral in internal florets. This pattern suggests that among all of the paralogs, the greatest difference in dorsoventral expression between external and internal florets is that of *KmCYC3B*. Additionally, *KmCYC3B* has as much of an increase in ventral expression in internal florets as *KmCYC2A*, the paralog with the highest expression in dorsal petals by a wide margin. Our evidence supports the need for additional studies on the role of CYC3 genes in floral symmetry.

## Conclusions

Shifts between radial symmetry and bilateral symmetry are correlated with the restriction of *CYCLOIDEA*-like genes to the dorsal (upper) region of the flower. Here, we quantify, for the first time, the differences in expression of these genes across petals from flowers with differing degrees of bilateral symmetry in the same species. We find that it is the ventral (lower) expression, although weak, that significantly changes between these two flower types. Therefore, it is not the seemingly specific expression of these genes in the dorsal region, but the difference in dorsoventral expression that likely governs symmetry. These data also suggest that changes in genes that are weakly expressed can have a large effect on flower shape.

## Methods

*Knautia macedonica* Griseb. plants were grown to flower at St. John’s University, Queens, NY, USA. Internal and external buds from immature capitula were collected or dissected into dorsal, left lateral, right lateral, and ventral petal types (i.e., corolla tube tissue excluded from dissections) at various stages of development up to anthesis and prior to pigment production. Because of limited petal tissue per floret, dissections were pooled from multiple accessions and different capitula to obtain 20–25 mg of tissue for RNA extraction. Leaf RNA was also collected for comparison. Total RNA was extracted using the RNeasy Plant Mini Kit, including the RNase-free DNase (Qiagen) on-column step. Sample concentrations and purities were determined using a Thermo Scientific NanoDrop 2000.

Total cDNA was generated using the Omniscript RT kit (Qiagen) following the standard protocol and including 4 µL of ~50 ng/µL concentration of each RNA sample. qPCR primers were designed in Geneious Pro v.6.1.8 (http://www.geneious.com; [45]) based on available *CYC*-like genes and GAPDH sequences (see Additional files 4, 5). Amplicons were confirmed via sequencing at the Yale DNA Analysis Facility. Six new *CYC*-like paralog sequences obtained of *K. macedonica* were combined with 159 *CYC*-like gene sequences downloaded from GenBank (Additional file 5) representing all major lineages of the broadly circumscribed clade Caprifoliaceae (including Dipsacaceae; [24]) for phylogenetic analyses. Alignments from [7, 14, 19, 24] were obtained via personal communication from D.G. Howarth and S.E. Carlson, and new sequences were added in Geneious Pro v.6.1.8 (http://www.geneious.com; [45]) using the Geneious Alignment tool (default parameters) based on nucleotide sequences, and non-Caprifoliaceae sequences were removed. Aligned nucleotide datasets were then manually adjusted based on amino acid codon sequence.

The best-fitting model of sequence evolution (TIM3 + I + G) was determined using the Bayesian information criterion (BIC) in jModeltest v.2.1.6 [46, 47]. ML and BI analyses incorporating the best model of sequence evolution were both conducted on the CIPRES Science Gateway v.3.3 (www.phylo.org) using RAxML v.8.1.24 [48] and MrBayes v.3.2.3 [49–51] (Additional file 1). ML analyses were conducted using default parameters to obtain the single best tree and 1000 BS replicates under the GTRGAMMA model (suggested by the developer of RAxML). A single CYC1 gene was initially used to root the tree in RAxML. Trees were read into PAUP* [52], derooted, and rerooted with all CYC1 genes used as the monophyletic outgroup for the CYC2 and CYC3 copies. Two BI analyses were run in parallel for 20,000,000 generations with four chains sampling every 2000 generations. Stationarity was determined using Tracer v.1.6 {Rambaut:-M7jROaJ}. Trees were read into PAUP* and 20 % were discarded as burn-in prior to constructing a 50 % majority rule consensus tree.

Primer efficiency was determined using a melting curve analysis [53, 54]. Samples were normalized to 20 ng/µL, and qPCR was performed using 20 µL reactions of the iTaq universal SYBR green one-step kit (BioRad) on a MyIQ (BioRad) machine. Relative expression levels were calculated using the 2^−∆∆CT^ method [55]. GAPDH was used as the reference gene based on preliminary data (Additional file 6) that revealed consistent expression levels regardless of the tissue type (i.e., leaf tissue vs. bud tissue vs. petal tissue). All samples were normalized to GAPDH expression, and three biological replicates were analyzed in duplicate. ANOVA and post hoc Tukey HSD tests were performed in R (R Development Core Team 2015; see Additional file 7). Plots were made using the ggplot2 package v.1.0.1 [56] as implemented in R.

## Additional files

10.1186/s13227-016-0045-7 Bayesian inference tree of *CYC*-like genes across Caprifoliaceae (including Dipsacaceae). Duplication events are designated with dark circles. Maximum likelihood bootstrap (BS) values and Bayesian inference posterior probabilities (PP) are shown above branches (ML-BS/BI-PP. Asterisks (*) denote BS values ≤50. Clade names follow [7, 19, 23]. Paralogs of *Knautia macedonica* examined in this study are highlighted in gray.
10.1186/s13227-016-0045-7 qPCR relative expression of *CYC*-like paralogs for external whole buds, internal whole buds, and leaf. Standard error bars are shown. Statistical significance among petal types is as follows: * = *p* ≤ 0.05, ** = *p* ≤ 0.01, *** = *p* ≤ 0.001.
10.1186/s13227-016-0045-7 qPCR relative expression of *CYC*-like paralogs for dorsal, left lateral, right lateral, and ventral petals of external and internal florets. Standard error bars are shown.
10.1186/s13227-016-0045-7 Primer sequences used for PCR and qPCR experiments. Product size obtained from *Knautia macedonica* is provided.
10.1186/s13227-016-0045-7 GenBank accession numbers for sequences included in phylogenetic reconstruction of *CYC*-like genes across Dipsacales.
10.1186/s13227-016-0045-7 Preliminary qPCR expression data of GAPDH across all tissue types. Quantification cycles are shown for three independent biological replicates of each tissue type. RNA concentrations for each sample were normalized prior to qPCR experiments (20 ng/reaction). EXT = external, INT = internal, D = dorsal petals, LL = left lateral petals, RL = right lateral petals, V = ventral petals.
10.1186/s13227-016-0045-7 ANOVA and Tukey HSD analyses performed in R.

## Abbreviations

ECE: glutamic acid–cysteine–glutamic acid
CYC: *CYCLOIDEA*
TCP: Teosinte Branched1, CYCLOIDEA, Proliferating cell factor
qPCR: quantitative real-time polymerase chain reaction
RT-PCR: reverse transcription polymerase chain reaction
ANOVA: analysis of variance
HSD: honest significant difference
CT: threshold cycle
