# Supplementary material for: Elaboration of bilateral symmetry across Knautia macedonica capitula related to changes in ventral petal expression of CYCLOIDEA-like genes
Source: EvoDevo. 2016 Mar 31;7:8. doi: 10.1186/s13227-016-0045-7 (PMC4818532; doi:10.1186/s13227-016-0045-7)
Supplement: Supplementary file 4 — 10.1186/s13227-016-0045-7 Primer sequences used for PCR and qPCR experiments. Product size obtained from Knautia macedonica is provided. [file 13227_2016_45_MOESM4_ESM.docx]

Additional file 4. Primers used for PCR and qPCR experiments. Product size obtained from *Knautia macedonica* is provided.

| Primer | Sequence (5’-3’) | Product size (bp) | Reference |
| --- | --- | --- | --- |
| KmGAPDHF | CAAGCAAAGATGCTCCGATG | 225 | This study |
| KmGAPDHR | CACCTCTCCAGTCCTTGCTC |  |  |
| KmCYC1F1 | TTACTCAATCAATCAAGATCTGCC | 248 | This study |
| KmCYC1R1 | AACTCCCTAGCAGTAGAGTCGAATGC |  |  |
| KmCYC2AF1 | ACAAGGCGAGTCAAACAC | 269 | This study |
| KmCYC2AR1 | CCTAGACTCTTTAGCTGCAAG |  |  |
| KmCYC2BaF1 | AAAGCAAGCAAAACCCTAGATTGGC | 156 | This study |
| KmCYC2BaR1 | AGAACTCGTACCAAATCCGTCCC |  |  |
| KmCYC2BbF1 | ACTGGATGCTTGTATGAAATGG | 106 | This study |
| KmCYC2BbR1 | TAGATTCTCGAGAACGCG |  |  |
| KmCYC3AF1 | AAGGCTATAAGAGAAGTAACCCTAAAACACCCAC | 160 | This study |
| KmCYC3AR1 | TAGCCTTGTTCCTCATCTCTCTTGCAACC |  |  |
| KmCYC3BF1 | ATCAAAAGGAGCAATTAAAGAAGTCACCAG | 86 | This study |
| KmCYC3BR1 | TTTGTTCTTTTTACCCTCGGATGAACC |  |  |
