# Supplementary material for: Elaboration of bilateral symmetry across Knautia macedonica capitula related to changes in ventral petal expression of CYCLOIDEA-like genes
Source: EvoDevo. 2016 Mar 31;7:8. doi: 10.1186/s13227-016-0045-7 (PMC4818532; doi:10.1186/s13227-016-0045-7)
Supplement: Supplementary file 5 — 10.1186/s13227-016-0045-7 GenBank accession numbers for sequences included in phylogenetic reconstruction of CYC-like genes across Dipsacales. [file 13227_2016_45_MOESM5_ESM.docx]

Additional file 5. GenBank accession numbers for *CYC*-like sequences included in this study.

|  | **CYC-like Genes** | | | | | | | | |  |
| --- | --- | --- | --- | --- | --- | --- | --- | --- | --- | --- |
| **Species** | **1** | **1A** | **1B** | **2A** | **2B** | **2Ba** | **2Bb** | **3A** | **3B** | **Reference** |
| *Abelia* × *grandiflora* | **AY85165** |  |  |  |  |  |  | **AY851222**  **AY851223** |  | 19 |
| *Bassecoia bretschneideri* | **JN944774** |  |  | **JN944790** |  | **JN944800** | **JN944822** |  | **JN944845** | 23 |
| *Centranthus ruber* |  |  |  | **AY851184** |  | **AY851185** | **AY851186** | **AY851224** | **AY851225** | 19 |
| *Cephalaria hirsuta* | **JN944776** |  | **JN944778** | **JN944792** |  | **JN944814**  **JN944818** | **JN944824** |  |  | 23 |
| *Cephalaria humilis* |  |  | **JN944782** | **JN944793** |  | **JN944807**  **JN944816** | **JN944831** |  |  | 23 |
| *Cryptothladia chinensis* |  |  |  |  |  | **AY851187** | **AY851188** | **AY851226** | **AY851227**  **AY851228** | 19 |
| *Cryptothladia kokonorica* |  |  |  | **AY851189** |  | **AY851190** | **AY851191** |  | **AY851229**  **AY851230** | 19 |
| *Diervilla sessilifolia* | **AY851166** |  |  | **AY851192** | **AY851193** |  |  | **AY851231** |  | 19 |
| *Dipelta floribunda* | **AY851167** |  |  | **AY851194** | **AY851195** |  |  | **AY851232** | **AY851233** | 19 |
| *Dipsacus inermis* | **JN944775** |  |  | **JN944791** |  | **JN944802** |  |  |  | 23 |
| *Dipsacus pilosus* | **AY851168** |  |  |  |  | **AY851196** | **AY851197** |  | **AY851234** | 19 |
| *Fedia cornucopiae* | **XXXXXX** |  |  | **XXXXXX** |  | **XXXXXX** | **XXXXXX** | **XXXXXX** | **XXXXXX** | This study |
| *Heptacodium miconioides* | **AY851169** |  |  |  | **AY851198** |  |  | **AY851235** | **AY851236** | 19 |
| *Knautia calycina* | **JN944777** |  |  | **JN944794** |  | **JN944803**  **JN944806**  **JN944813** | **JN944825**  **JN944832** |  | **JN944842**  **JN944843** | 23 |
| *Knautia macedonica* | **XXXXXX** |  |  | **XXXXXX** |  | **XXXXXX** | **XXXXXX** | **XXXXXX** | **XXXXXX** | This study |
| *Kolkwitzia amabilis* | **AY851170** |  |  | **AY851199** | **AY851200** |  |  | **AY851237** | **AY851238** | 19 |
| *Leycesteria formosa* | **AY851171** |  |  | **AY851201** |  |  |  |  |  | 19 |
| *Leycesteria* sp. | **AY851172** |  |  |  |  |  |  |  |  | 19 |
| *Linnaea borealis* | **AY851172** |  |  |  |  |  |  | **AY851239** |  | 19 |
| *Lomelosia crenata* |  |  | **JN944784** | **JN944799** |  | **JN944805**  **JN944817** | **JN944830** |  |  | 23 |
| *Lonicera heteroloba* | **AY851174** |  |  | **AY851202** | **AY851203** |  |  | **AY851240**  **AY851241** |  | 19 |
| *Lonicera reticulata (=L. prolifera)* |  |  |  | **AY851204** |  |  |  | **AY851242** | **AY851243** | 19 |
| *Morina longifolia* |  |  |  |  |  | **AY851205** | **AY851206** |  | **AY851244**  **AY851245** | 19 |
| *Patrinia triloba* | **AY851175** |  |  | **AY851207** | **AY851208** |  |  |  | **AY851246** | 19 |
| *Pseudoscabiosa limonifolia* |  |  |  |  |  | **JN944801** | **JN944823** |  | **JN944844** | 23 |
| *Pterocephalus strictus* |  |  | **JN944785**  **JN944789** | **JN944797** |  | **JN944809**  **JN944810**  **JN944821** | **JN944828**  **JN944833** | **JN944838** | **JN944841** | 23 |
| *Pycnocomon rutifolium* |  |  | **JN944781** | **JN944798** |  | **JN944812** | **JN944829**  **JN944834** | **JN944839** |  | 23 |
| *Scabiosa angustiloba* |  |  | **JN944779**  **JN944786** | **JN944795** |  | **JN944811** | **JN944826** | **JN944836** |  | 23 |
| *Scabiosa columbaria* | **AY851177** |  |  |  |  |  |  |  |  | 19 |
| *Sixalix atropurpurea* |  |  | **JN944780**  **JN944783**  **JN944787**  **JN944788** | **JN944796** |  | **JN944804**  **JN944815**  **JN944819**  **JN944820** | **JN944827** | **JN944837** | **JN944840** | 23 |
| *Sixalix farinosa* |  |  |  |  |  | **JN944808** | **JN944835** |  |  | 23 |
| *Symphoricarpos occidentalis* | **AY851178** |  |  |  |  |  |  | **AY851249** | **AY851250** | 19 |
| *Symphoricarpos*  *orbiculatus* | **AY851179** |  |  | **AY851210** |  |  |  |  |  | 19 |
| *Triosteum himalayanum* | **AY851180** |  |  | **AY851211** | **AY851212** |  |  | **AY851251** |  | 19 |
| *Triplostegia glandulifera* | **AY851181** |  |  |  | **AY851213** |  |  |  | **AY851252** | 19 |
| *Valerianlla dentata* | **AY851182** |  |  |  | **AY851214** |  |  |  | **AY851253** | 19 |
| *Weigela hortensis* | **AY851183** |  |  | **AY851220** | **AY851221** |  |  | **AY851255** |  | 19 |
