# Supplementary material for: Elaboration of bilateral symmetry across Knautia macedonica capitula related to changes in ventral petal expression of CYCLOIDEA-like genes
Source: EvoDevo. 2016 Mar 31;7:8. doi: 10.1186/s13227-016-0045-7 (PMC4818532; doi:10.1186/s13227-016-0045-7)
Supplement: Supplementary file 6 — 10.1186/s13227-016-0045-7 Preliminary qPCR expression data of GAPDH across all tissue types. Quantification cycles are shown for three independent biological replicates of each tissue type. RNA concentrations for each sample were normalized prior to qPCR experiments (20 ng/reaction). EXT = external, INT = internal, D = dorsal petals, LL = left lateral petals, RL = right lateral petals, V = ventral petals. [file 13227_2016_45_MOESM6_ESM.docx]

| GAPDH | Leaf | EXT buds | EXT D | EXT LL | EXT RL | EXT V | INT buds | INT D | INT LL | INT RL | INT V |
| --- | --- | --- | --- | --- | --- | --- | --- | --- | --- | --- | --- |
| Rep1 | 20.9 | 20.9 | 20.7 | 20.7 | 20.9 | 20.8 | 21.2 | 21.1 | 21.2 | 20.1 | 20.4 |
| Rep2 | 20.8 | 20.8 | 20.6 | 20.5 | 20.6 | 21.1 | 20.8 | 20.9 | 20.9 | 21.0 | 20.5 |
| Rep3 | 20.4 | 20.5 | 20.5 | 20.8 | 20.7 | 20.0 | 20.9 | 20.6 | 20.3 | 20.6 | 20.6 |

Additional file 6. Preliminary qPCR expression data of GAPDH across all tissue types. Quantification cycles are shown for three independent biological replicates of each tissue type. RNA concentrations for each sample were normalized prior to qPCR experiments (20ng/reaction). EXT = external, INT = internal, D = dorsal petals, LL = left lateral petals, RL = right lateral petals, V = ventral petals.
