# Supplementary material for: Elaboration of bilateral symmetry across Knautia macedonica capitula related to changes in ventral petal expression of CYCLOIDEA-like genes
Source: EvoDevo. 2016 Mar 31;7:8. doi: 10.1186/s13227-016-0045-7 (PMC4818532; doi:10.1186/s13227-016-0045-7)
Supplement: Supplementary file 7 — 10.1186/s13227-016-0045-7 ANOVA and Tukey HSD analyses performed in R. [file 13227_2016_45_MOESM7_ESM.pdf]

Additional file 7. Data obtained from ANOVA and post-hoc Tukey tests.

```
##########################################################################################
##########################################################################################

ANOVA for CYC1 Comparing INT and EXT buds.

              Df Sum Sq Mean Sq F value Pr(F)
CYC1.b$flower  1 0.4555  0.4555   8.893 0.0407 *
Residuals      4 0.2049  0.0512

Signif. codes:  0 ‘***’ 0.001 ‘**’ 0.01 ‘*’ 0.05 ‘.’ 0.1 ‘ ’ 1

  Tukey multiple comparisons of means
    95% family-wise confidence level

CYC1.b$flower
                       diff      lwr         upr     p adj
internal-external -0.551052 -1.06411 -0.03799405 0.0406564
--
ANOVA for CYC2A Comparing INT and EXT buds.

               Df Sum Sq Mean Sq F value Pr(F)
CYC2A.b$flower  1 0.3905  0.3905   12.73 0.0118 *
Residuals       6 0.1840  0.0307

Signif. codes:  0 ‘***’ 0.001 ‘**’ 0.01 ‘*’ 0.05 ‘.’ 0.1 ‘ ’ 1

  Tukey multiple comparisons of means
    95% family-wise confidence level

CYC2A.b$flower
                        diff        lwr        upr    p adj
internal-external -0.4418814 -0.7448635 -0.1388992 0.011803
--
ANOVA for CYC2Ba Comparing INT and EXT buds.

                 Df Sum Sq Mean Sq F value Pr(F)
CYC2Ba.b$flower  1 0.5657  0.5657   3.894 0.0839 .
Residuals         8 1.1622  0.1453

Signif. codes:  0 ‘***’ 0.001 ‘**’ 0.01 ‘*’ 0.05 ‘.’ 0.1 ‘ ’ 1

  Tukey multiple comparisons of means
    95% family-wise confidence level

CYC2Ba.b$flower
                       diff         lwr      upr     p adj
internal-external 0.4854918 -0.08186518 1.052849 0.0839186
--
ANOVA for CYC2Bb Comparing INT and EXT buds.

                Df Sum Sq Mean Sq F value  Pr(F)
CYC2Bb.b$flower  1 1.0468  1.0468   12.66 0.00449 **
Residuals       11 0.9096  0.0827

Signif. codes:  0 ‘***’ 0.001 ‘**’ 0.01 ‘*’ 0.05 ‘.’ 0.1 ‘ ’ 1

  Tukey multiple comparisons of means
    95% family-wise confidence level

CYC2Bb.b$flower
                       diff       lwr       upr     p adj
internal-external 0.5832833 0.2224651 0.9441015 0.0044884
--
ANOVA for CYC3A Comparing INT and EXT buds.

               Df Sum Sq Mean Sq F value Pr(F)
```

```
CYC3A.b$flower  1 0.5004  0.5004   7.679 0.0243 *
Residuals       8 0.5213  0.0652

Signif. codes:  0 '***' 0.001 '**' 0.01 '*' 0.05 '.' 0.1 ' ' 1

  Tukey multiple comparisons of means
    95% family-wise confidence level

CYC3A.b$flower
                       diff        lwr       upr     p adj
internal-external 0.4566105 0.07664512 0.8365758 0.0242531
--
ANOVA for CYC3B Comparing INT and EXT buds.

                Df  Sum Sq Mean Sq F value Pr(F)
CYC3B.b$flower  1 0.04872 0.04872   1.564  0.279
Residuals        4 0.12460 0.03115

  Tukey multiple comparisons of means
    95% family-wise confidence level

CYC3B.b$flower
                        diff        lwr       upr     p adj
internal-external -0.1802183 -0.5803195 0.2198829 0.2792411

##########################################################################################
##########################################################################################

ANOVA Expression Comparisons of CYC by Flower Position (INTERNAL vs. EXTERNAL).

Dorsal Petal ANOVA Expression Comparisons of CYC1.

             Df Sum Sq Mean Sq F value Pr(>F)
CYC1d$flower  1  2.462  2.4625   3.225  0.123
Residuals     6  4.581  0.7635

  Tukey multiple comparisons of means
    95% family-wise confidence level

CYC1d$flower
                       diff       lwr       upr     p adj
internal-external -1.146003 -2.707465 0.4154594 0.1226536
--
Dorsal Petal ANOVA Expression Comparisons of CYC2A.

              Df  Sum Sq Mean Sq F value Pr(>F)
CYC2Ad$flower  1 0.04041 0.04041   2.184  0.174
Residuals      9 0.16657 0.01851

  Tukey multiple comparisons of means
    95% family-wise confidence level

CYC2Ad$flower
                        diff        lwr        upr     p adj
internal-external -0.1260043 -0.3188984 0.06688987 0.1736041
--
Dorsal Petal ANOVA Expression Comparisons of CYC2Ba.

                Df Sum Sq Mean Sq F value Pr(>F)
CYC2Bad$flower  1 0.0058 0.00577    0.02  0.892
Residuals       7 2.0219 0.28884

  Tukey multiple comparisons of means
    95% family-wise confidence level

CYC2Bad$flower
                        diff       lwr       upr     p adj
internal-external -0.0537189 -0.952334 0.8448962 0.8915705
```

```
—
Dorsal Petal ANOVA Expression Comparisons of CYC2Bb.

               Df Sum Sq Mean Sq F value Pr(>F)
CYC2Bbd$flower  1  0.445  0.4447   1.134  0.302
Residuals      17  6.666  0.3921

  Tukey multiple comparisons of means
    95% family-wise confidence level

CYC2Bbd$flower
                        diff        lwr       upr     p adj
internal-external -0.3098683 -0.9237387 0.3040021 0.3017744
—
Dorsal Petal ANOVA Expression Comparisons of CYC3A.

              Df Sum Sq Mean Sq F value Pr(>F)
CYC3Ad$flower  1  2.142  2.1416   3.137  0.102
Residuals     12  8.193  0.6828

  Tukey multiple comparisons of means
    95% family-wise confidence level

CYC3Ad$flower
                       diff        lwr      upr     p adj
internal-external 0.7903324 -0.1819567 1.762621 0.1019222
—
Dorsal Petal ANOVA Expression Comparisons of CYC3B.

               Df Sum Sq Mean Sq F value Pr(>F)
CYC3Bd$flower  1 0.0744 0.07444   0.979  0.355
Residuals      7 0.5323 0.07604

  Tukey multiple comparisons of means
    95% family-wise confidence level

CYC3Bd$flower
                       diff        lwr       upr    p adj
internal-external -0.192926 -0.6539861 0.2681341 0.355402

##########################################################################################
##########################################################################################

ANOVA Expression Comparisons of CYC by Flower Position (INTERNAL vs. EXTERNAL).

Left Lateral Petal ANOVA Expression Comparisons of CYC1.

              Df Sum Sq Mean Sq F value Pr(>F)
CYC1LL$flower  1  1.614  1.6144   3.521   0.09 .
Residuals     10  4.585  0.4585

Signif. codes:  0 ‘***’ 0.001 ‘**’ 0.01 ‘*’ 0.05 ‘.’ 0.1 ‘ ’ 1

  Tukey multiple comparisons of means
    95% family-wise confidence level

CYC1LL$flower
                       diff       lwr      upr     p adj
internal-external 0.7335807 -0.137491 1.604652 0.0900485

—
Left Lateral Petal ANOVA Expression Comparisons of CYC2A.

               Df Sum Sq Mean Sq F value Pr(>F)
CYC2ALL$flower  1   0.06  0.0601   0.094  0.762
Residuals      19  12.13  0.6382

  Tukey multiple comparisons of means
```

```
    95% family-wise confidence level

CYC2ALL$flower
                       diff        lwr       upr    p adj
internal-external 0.1081117 -0.6292218 0.8454452 0.762265

__
Left Lateral Petal ANOVA Expression Comparisons of CYC2Ba.

                 Df Sum Sq Mean Sq F value Pr(>F)
CYC2Ba1LL$flower  1  0.217  0.2166   0.408  0.534
Residuals        13  6.898  0.5306

  Tukey multiple comparisons of means
    95% family-wise confidence level

CYC2BaLL$flower
                       diff       lwr       upr     p adj
internal-external -0.245297 -1.074693 0.5840992 0.5339601

__
Left Lateral Petal ANOVA Expression Comparisons of CYC2Bb.

                Df Sum Sq Mean Sq F value  Pr(>F)
CYC2BbLL$flower  1 0.5751  0.5751   12.08 0.00596 **
Residuals       10 0.4761  0.0476
---
Signif. codes:  0 ‘***’ 0.001 ‘**’ 0.01 ‘*’ 0.05 ‘.’ 0.1 ‘ ’ 1

  Tukey multiple comparisons of means
    95% family-wise confidence level

CYC2BbLL$flower
                       diff        lwr        upr     p adj
internal-external -0.437838 -0.7185189 -0.1571571 0.0059639

__
Left Lateral Petal ANOVA Expression Comparisons of CYC3A.

              Df Sum Sq Mean Sq F value Pr(>F)
CYC3ALL$flower  1  0.692  0.6915   1.024  0.345
Residuals       7  4.726  0.6751

  Tukey multiple comparisons of means
    95% family-wise confidence level

CYC3ALL$flower
                        diff       lwr       upr     p adj
internal-external -0.5880201 -1.961821 0.7857812 0.3451811

__
Left Lateral Petal ANOVA Expression Comparisons of CYC3B.

                Df Sum Sq Mean Sq F value Pr(>F)
CYC3BLL$flower  1  0.181  0.1814    0.43  0.524
Residuals      13  5.490  0.4223

  Tukey multiple comparisons of means
    95% family-wise confidence level

CYC3BLL$flower
                        diff        lwr       upr     p adj
internal-external -0.2245027 -0.9644067 0.5154014 0.5235761

##########################################################################################
##########################################################################################

Right Lateral Petal ANOVA Expression Comparisons based on INTERNAL vs. EXTERNAL Flower Position
```

```
Right Lateral Petal ANOVA Expression Comparisons of CYC1.

              Df Sum Sq Mean Sq F value Pr(>F)
CYC1RL$flower  1  0.001  0.0005   0.001  0.978
Residuals      8  5.190  0.6488

  Tukey multiple comparisons of means
    95% family-wise confidence level

CYC1RL$flower
                         diff       lwr      upr     p adj
internal-external -0.01472943 -1.213688 1.184229 0.9780932

---
Right Lateral Petal ANOVA Expression Comparisons of CYC2A.

               Df Sum Sq Mean Sq F value Pr(>F)
CYC2ARL$flower  1  1.220  1.2204   1.851  0.197
Residuals      13  8.574  0.6595

  Tukey multiple comparisons of means
    95% family-wise confidence level

CYC2ARL$flower
                        diff       lwr       upr     p adj
internal-external -0.5822436 -1.506911 0.3424235 0.1968445

---
Right Lateral Petal ANOVA Expression Comparisons of CYC2Ba.

                 Df Sum Sq Mean Sq F value Pr(>F)
CYC2BaRL$flower  1  2.707  2.7066   3.649 0.0722 .
Residuals        18 13.350  0.7417

Signif. codes:  0 ‘***’ 0.001 ‘**’ 0.01 ‘*’ 0.05 ‘.’ 0.1 ‘ ’ 1

  Tukey multiple comparisons of means
    95% family-wise confidence level

CYC2BaRL$flower
                        diff       lwr        upr     p adj
internal-external -0.7509095 -1.576743 0.07492351 0.0721568

---
Right Lateral Petal ANOVA Expression Comparisons of CYC2Bb.

                Df Sum Sq Mean Sq F value Pr(>F)
CYC2BbRL$flower  1 0.7318  0.7318   3.724 0.0897 .
Residuals        8 1.5720  0.1965

Signif. codes:  0 ‘***’ 0.001 ‘**’ 0.01 ‘*’ 0.05 ‘.’ 0.1 ‘ ’ 1

  Tukey multiple comparisons of means
    95% family-wise confidence level

CYC2BbRL$flower
                        diff       lwr       upr     p adj
internal-external -0.5903304 -1.295719 0.1150587 0.0897396

---
Right Lateral Petal ANOVA Expression Comparisons of CYC3A.

               Df Sum Sq Mean Sq F value Pr(>F)
CYC3ARL$flower  1  3.557   3.557   9.642  0.036 *
Residuals       4  1.476   0.369

Signif. codes:  0 ‘***’ 0.001 ‘**’ 0.01 ‘*’ 0.05 ‘.’ 0.1 ‘ ’ 1
```

```
  Tukey multiple comparisons of means
    95% family-wise confidence level

CYC3ARL$flower
                      diff       lwr      upr     p adj
internal-external 1.539941 0.1629933 2.916889 0.0360427

__
Right Lateral Petal ANOVA Expression Comparisons of CYC3B.

                Df Sum Sq Mean Sq F value Pr(>F)
CYC3BRL$flower  1  0.326  0.3256   0.567  0.466
Residuals      12  6.893  0.5744

  Tukey multiple comparisons of means
    95% family-wise confidence level

CYC3BRL$flower
                       diff        lwr      upr     p adj
internal-external 0.3081617 -0.5836476 1.199971 0.4660352

##########################################################################################
##########################################################################################

ANOVA Expression Comparisons of CYC by Flower Position (INTERNAL vs. EXTERNAL).

Ventral Lateral Petal ANOVA Expression Comparisons of CYC1.

             Df Sum Sq Mean Sq F value Pr(>F)
CYC1v$flower  1  4.171   4.171   5.293 0.0549 .
Residuals     7  5.515   0.788

Signif. codes:  0 ‘***’ 0.001 ‘**’ 0.01 ‘*’ 0.05 ‘.’ 0.1 ‘ ’ 1

  Tukey multiple comparisons of means
    95% family-wise confidence level

CYC1v$flower
                      diff         lwr      upr     p adj
internal-external 1.444057 -0.04012891 2.928243 0.0549352

__
Ventral Petal ANOVA Expression Comparisons of CYC2A.

              Df Sum Sq Mean Sq F value Pr(>F)
CYC2Av$flower  1   6.72   6.720   5.186 0.0403 *
Residuals     13  16.85   1.296

Signif. codes:  0 ‘***’ 0.001 ‘**’ 0.01 ‘*’ 0.05 ‘.’ 0.1 ‘ ’ 1

  Tukey multiple comparisons of means
    95% family-wise confidence level

CYC2Av$flower
                     diff        lwr     upr    p adj
internal-external 1.36627 0.07013065 2.66241 0.040323

__
Ventral Petal ANOVA Expression Comparisons of CYC2Ba.

                Df Sum Sq Mean Sq F value  Pr(>F)
CYC2Bav$flower  1  3.059  3.0588   18.03 0.00381 **
Residuals        7  1.188  0.1697

Signif. codes:  0 ‘***’ 0.001 ‘**’ 0.01 ‘*’ 0.05 ‘.’ 0.1 ‘ ’ 1

  Tukey multiple comparisons of means
```

```
    95% family-wise confidence level

CYC2Bav$flower
                     diff       lwr      upr     p adj
internal-external 1.23669 0.5479473 1.925433 0.0038126
```

__

Ventral Petal ANOVA Expression Comparisons of CYC2Bb.

```
               Df Sum Sq Mean Sq F value Pr(>F)
CYC2Bbv$flower  1  0.317  0.3169   0.512  0.487
Residuals      13  8.054  0.6195

  Tukey multiple comparisons of means
    95% family-wise confidence level

CYC2Bbv$flower
                       diff        lwr     upr     p adj
internal-external 0.2967057 -0.5995081 1.19292 0.4871178
```

__

Ventral Petal ANOVA Expression Comparisons of CYC3A.

```
              Df Sum Sq Mean Sq F value Pr(>F)
CYC3Av$flower  1  0.266  0.2660   1.619  0.227
Residuals     12  1.971  0.1643

  Tukey multiple comparisons of means
    95% family-wise confidence level

CYC3Av$flower
                       diff        lwr       upr     p adj
internal-external 0.2876559 -0.2048907 0.7802025 0.2273112
```

__

Ventral Petal ANOVA Expression Comparisons of CYC3B.

```
               Df Sum Sq Mean Sq F value Pr(>F)
CYC3Bbv$flower  1  2.069  2.0694   11.36 0.0119 *
Residuals       7  1.275  0.1821

Signif. codes:  0 ‘***’ 0.001 ‘**’ 0.01 ‘*’ 0.05 ‘.’ 0.1 ‘ ’ 1

  Tukey multiple comparisons of means
    95% family-wise confidence level

CYC3Bbv$flower
                      diff       lwr      upr     p adj
internal-external 1.017193 0.3036444 1.730743 0.0119062
```

##
##

Gene comparisons based on petal in internal and external florets.

Internal dorsal petal comparisons.

```
              Df Sum Sq Mean Sq F value   Pr(>F)
CYC1.i.d$gene  5  59.06  11.813   29.84 7.28e-09 ***
Residuals     21   8.31   0.396

Signif. codes:  0 ‘***’ 0.001 ‘**’ 0.01 ‘*’ 0.05 ‘.’ 0.1 ‘ ’ 1

  Tukey multiple comparisons of means
    95% family-wise confidence level

CYC1.i.d$gene
                     diff        lwr         upr     p adj
```

```
CYC2A-CYC1      4.4505819  2.9471230  5.95404077 0.0000001
CYC2Ba-CYC1     1.2002992 -0.4069659  2.80756437 0.2238242
CYC2Bb-CYC1     3.1751911  1.8425172  4.50786495 0.0000034
CYC3A-CYC1      0.9213074 -0.4706250  2.31323985 0.3390091
CYC3B-CYC1      3.3860233  1.7787582  4.99328849 0.0000210
CYC2Ba-CYC2A   -3.2502827 -4.7537415 -1.74682380 0.0000145
CYC2Bb-CYC2A   -1.2753908 -2.4808397 -0.06994196 0.0341217
CYC3A-CYC2A    -3.5292745 -4.7999292 -2.25861984 0.0000003
CYC3B-CYC2A    -1.0645586 -2.5680174  0.43890031 0.2723212
CYC2Bb-CYC2Ba   1.9748919  0.6422180  3.30756571 0.0017269
CYC3A-CYC2Ba   -0.2789918 -1.6709243  1.11294061 0.9876528
CYC3B-CYC2Ba    2.1857241  0.5784590  3.79298925 0.0041536
CYC3A-CYC2Bb   -2.2538837 -3.3169896 -1.19077773 0.0000192
CYC3B-CYC2Bb    0.2108323 -1.1218416  1.54350610 0.9958306
CYC3B-CYC3A     2.4647159  1.0727835  3.85664838 0.0002174
```

External dorsal petal comparisons.

```
              Df Sum Sq Mean Sq F value Pr(>F)
CYC1.e.d$gene  5 111.18  22.236   59.42 <2e-16 ***
Residuals     37  13.85   0.374

Signif. codes:  0 ‘***’ 0.001 ‘**’ 0.01 ‘*’ 0.05 ‘.’ 0.1 ‘ ’ 1

  Tukey multiple comparisons of means
    95% family-wise confidence level

CYC1.e.d$gene
                     diff       lwr         upr     p adj
CYC2A-CYC1      3.43058340  2.354500  4.50666708 0.0000000
CYC2Ba-CYC1     0.10801538 -1.004805  1.22083599 0.9996914
CYC2Bb-CYC1     2.33905664  1.347841  3.33027215 0.0000003
CYC3A-CYC1     -1.01502770 -2.062714  0.03265826 0.0621971
CYC3B-CYC1      2.43294656  1.320126  3.54576717 0.0000015
CYC2Ba-CYC2A   -3.32256802 -4.345005 -2.30013122 0.0000000
CYC2Bb-CYC2A   -1.09152677 -1.980074 -0.20297991 0.0086815
CYC3A-CYC2A    -4.44561110 -5.396744 -3.49447852 0.0000000
CYC3B-CYC2A    -0.99763684 -2.020074  0.02479996 0.0592862
CYC2Bb-CYC2Ba   2.23104126  1.298341  3.16374117 0.0000002
CYC3A-CYC2Ba   -1.12304308 -2.115548 -0.13053777 0.0187868
CYC3B-CYC2Ba    2.32493118  1.263898  3.38596399 0.0000015
CYC3A-CYC2Bb   -3.35408434 -4.208020 -2.50014915 0.0000000
CYC3B-CYC2Bb    0.09388993 -0.838810  1.02658984 0.9996312
CYC3B-CYC3A     3.44797426  2.455469  4.44047958 0.0000000
```

Internal left lateral petal comparisons.

```
               Df Sum Sq Mean Sq F value   Pr(>F)
CYC1.i.LL$gene  5  38.10   7.621   16.63 7.21e-08 ***
Residuals      30  13.75   0.458

Signif. codes:  0 ‘***’ 0.001 ‘**’ 0.01 ‘*’ 0.05 ‘.’ 0.1 ‘ ’ 1

  Tukey multiple comparisons of means
    95% family-wise confidence level

CYC1.i.LL$gene
                     diff        lwr        upr     p adj
CYC2A-CYC1      0.78075329 -0.3043485  1.8658550 0.2725628
CYC2Ba-CYC1     0.41877647 -0.7698929  1.6074459 0.8887175
CYC2Bb-CYC1     0.87567593 -0.3129935  2.0643453 0.2497270
CYC3A-CYC1     -3.02269959 -4.4785163 -1.5668828 0.0000081
CYC3B-CYC1      0.55047440 -0.6381950  1.7391438 0.7216859
CYC2Ba-CYC2A   -0.36197682 -1.4470786  0.7231249 0.9093775
CYC2Bb-CYC2A    0.09492265 -0.9901791  1.1800244 0.9997989
CYC3A-CYC2A    -3.80345288 -5.1760101 -2.4308957 0.0000000
CYC3B-CYC2A    -0.23027889 -1.3153806  0.8548228 0.9864080
CYC2Bb-CYC2Ba   0.45689947 -0.7317699  1.6455689 0.8477251
```

```
CYC3A-CYC2Ba   -3.44147606 -4.8972928 -1.9856593 0.0000008
CYC3B-CYC2Ba    0.13169793 -1.0569715  1.3203673 0.9993624
CYC3A-CYC2Bb   -3.89837552 -5.3541923 -2.4425588 0.0000001
CYC3B-CYC2Bb   -0.32520154 -1.5138709  0.8634679 0.9590535
CYC3B-CYC3A     3.57317399  2.1173572  5.0289907 0.0000004

External left lateral petal comparisons.

               Df Sum Sq Mean Sq F value   Pr(>F)    
CYC1.e.LL$gene  5  61.91  12.382    25.3 1.12e-11 ***
Residuals      42  20.56   0.489                     

Signif. codes:  0 ‘***’ 0.001 ‘**’ 0.01 ‘*’ 0.05 ‘.’ 0.1 ‘ ’ 1

  Tukey multiple comparisons of means
    95% family-wise confidence level

CYC1.e.LL$gene
                    diff        lwr        upr     p adj
CYC2A-CYC1      1.406222257  0.3620167  2.4504278 0.0030374
CYC2Ba-CYC1     1.397654173  0.2969649  2.4983434 0.0059121
CYC2Bb-CYC1     2.047094581  0.8413499  3.2528393 0.0001186
CYC3A-CYC1     -1.701098772 -2.9068435 -0.4953541 0.0017172
CYC3B-CYC1      1.508557725  0.4078684  2.6092470 0.0024602
CYC2Ba-CYC2A   -0.008568084 -0.9294708  0.9123346 1.0000000
CYC2Bb-CYC2A    0.640872324 -0.4033332  1.6850779 0.4569911
CYC3A-CYC2A    -3.107321028 -4.1515266 -2.0631155 0.0000000
CYC3B-CYC2A     0.102335468 -0.8185673  1.0232382 0.9994269
CYC2Bb-CYC2Ba   0.649440408 -0.4512489  1.7501297 0.5007367
CYC3A-CYC2Ba   -3.098752945 -4.1994422 -1.9980637 0.0000000
CYC3B-CYC2Ba    0.110903552 -0.8735829  1.0953900 0.9993876
CYC3A-CYC2Bb   -3.748193352 -4.9539380 -2.5424487 0.0000000
CYC3B-CYC2Bb   -0.538536856 -1.6392261  0.5621524 0.6903966
CYC3B-CYC3A     3.209656497  2.1089672  4.3103458 0.0000000

Internal right lateral petal comparisons.

               Df Sum Sq Mean Sq F value Pr(>F)  
CYC1.i.RL$gene  5  10.46  2.0918   3.038 0.0273 *
Residuals      26  17.90  0.6886                 

Signif. codes:  0 ‘***’ 0.001 ‘**’ 0.01 ‘*’ 0.05 ‘.’ 0.1 ‘ ’ 1

  Tukey multiple comparisons of means
    95% family-wise confidence level

CYC1.i.RL$gene
                   diff        lwr       upr     p adj
CYC2A-CYC1      0.46931844 -1.1764312 2.1150680 0.9488907
CYC2Ba-CYC1     0.20881057 -1.3524846 1.7701057 0.9983151
CYC2Bb-CYC1     0.91639835 -1.0308788 2.8636755 0.6997465
CYC3A-CYC1     -1.03019022 -2.9774674 0.9170870 0.5900083
CYC3B-CYC1      0.96867323 -0.5926219 2.5299684 0.4208777
CYC2Ba-CYC2A   -0.26050787 -1.6374408 1.1164250 0.9914354
CYC2Bb-CYC2A    0.44707991 -1.3557484 2.2499083 0.9715211
CYC3A-CYC2A    -1.49950866 -3.3023370 0.3033197 0.1448735
CYC3B-CYC2A     0.49935479 -0.8775781 1.8762877 0.8710565
CYC2Bb-CYC2Ba   0.70758778 -1.0184890 2.4336645 0.8034868
CYC3A-CYC2Ba   -1.23900079 -2.9650775 0.4870759 0.2692155
CYC3B-CYC2Ba    0.75986266 -0.5149295 2.0346548 0.4642990
CYC3A-CYC2Bb   -1.94658857 -4.0283154 0.1351383 0.0767325
CYC3B-CYC2Bb    0.05227488 -1.6738019 1.7783516 0.9999989
CYC3B-CYC3A     1.99886345  0.2727867 3.7249402 0.0164047

External right lateral petal comparisons.

               Df Sum Sq Mean Sq F value   Pr(>F)    
CYC1.e.RL$gene  5  41.27   8.254   15.95 2.27e-08 ***
```

```
Residuals      37  19.15   0.518

Signif. codes:  0 ‘***’ 0.001 ‘**’ 0.01 ‘*’ 0.05 ‘.’ 0.1 ‘ ’ 1

  Tukey multiple comparisons of means
    95% family-wise confidence level

CYC1.e.RL$gene
                     diff        lwr        upr     p adj
CYC2A-CYC1      1.03683263 -0.1022639  2.1759292 0.0922423
CYC2Ba-CYC1     0.94499064 -0.1356512  2.0256325 0.1160027
CYC2Bb-CYC1     1.49199927  0.2895720  2.6944266 0.0078455
CYC3A-CYC1     -2.58486060 -4.1131190 -1.0566022 0.0001508
CYC3B-CYC1      0.64578210 -0.6020357  1.8935999 0.6322537
CYC2Ba-CYC2A   -0.09184199 -1.0448786  0.8611946 0.9997021
CYC2Bb-CYC2A    0.45516663 -0.6340180  1.5443513 0.8065604
CYC3A-CYC2A    -3.62169323 -5.0625491 -2.1808374 0.0000001
CYC3B-CYC2A    -0.39105053 -1.5301471  0.7480460 0.9042934
CYC2Bb-CYC2Ba   0.54700863 -0.4808868  1.5749041 0.6045361
CYC3A-CYC2Ba   -3.52985124 -4.9249539 -2.1347486 0.0000001
CYC3B-CYC2Ba   -0.29920854 -1.3798504  0.7814333 0.9595965
CYC3A-CYC2Bb   -4.07685987 -5.5682874 -2.5854324 0.0000000
CYC3B-CYC2Bb   -0.84621717 -2.0486445  0.3562101 0.3024747
CYC3B-CYC3A     3.23064270  1.7023843  4.7589011 0.0000030

Internal ventral petal comparisons.

              Df Sum Sq Mean Sq F value Pr(>F)
CYC1.i.v$gene  5  8.836   1.767   3.133   0.03 *
Residuals     20 11.280   0.564

Signif. codes:  0 ‘***’ 0.001 ‘**’ 0.01 ‘*’ 0.05 ‘.’ 0.1 ‘ ’ 1

  Tukey multiple comparisons of means
    95% family-wise confidence level

CYC1.i.v$gene
                     diff        lwr       upr     p adj
CYC2A-CYC1      0.60072753 -1.0684878 2.2699429 0.8626564
CYC2Ba-CYC1    -0.03927834 -1.9667222 1.8881655 0.9999998
CYC2Bb-CYC1    -0.10339452 -1.7726098 1.5658208 0.9999545
CYC3A-CYC1     -0.69545768 -2.4194159 1.0285005 0.7982573
CYC3B-CYC1      1.23149138 -0.6959525 3.1589352 0.3721389
CYC2Ba-CYC2A   -0.64000586 -2.3092212 1.0292095 0.8292711
CYC2Bb-CYC2A   -0.70412204 -2.0670307 0.6587866 0.5933064
CYC3A-CYC2A    -1.29618520 -2.7256158 0.1332454 0.0896029
CYC3B-CYC2A     0.63076386 -1.0384515 2.2999792 0.8374443
CYC2Bb-CYC2Ba  -0.06411618 -1.7333315 1.6050991 0.9999958
CYC3A-CYC2Ba   -0.65617934 -2.3801375 1.0677788 0.8334184
CYC3B-CYC2Ba    1.27076972 -0.6566741 3.1982136 0.3398192
CYC3A-CYC2Bb   -0.59206316 -2.0214938 0.8373675 0.7806187
CYC3B-CYC2Bb    1.33488590 -0.3343294 3.0041012 0.1669699
CYC3B-CYC3A     1.92694906  0.2029909 3.6509072 0.0230031

External ventral petal comparisons.

              Df Sum Sq Mean Sq F value  Pr(>F)
CYC1.e.v$gene  5  11.71  2.3428   3.877 0.00601 **
Residuals     39  23.57  0.6043

Signif. codes:  0 ‘***’ 0.001 ‘**’ 0.01 ‘*’ 0.05 ‘.’ 0.1 ‘ ’ 1

  Tukey multiple comparisons of means
    95% family-wise confidence level

CYC1.e.v$gene
                    diff         lwr       upr     p adj
CYC2A-CYC1      0.6785144 -0.54899237 1.9060212 0.5678632
```

```
CYC2Ba-CYC1     0.1680884 -1.17657788 1.5127548 0.9989624
CYC2Bb-CYC1     1.0439568 -0.18355001 2.2714636 0.1352304
CYC3A-CYC1      0.4609435 -0.76656330 1.6884503 0.8679930
CYC3B-CYC1      1.6583550  0.31368867 3.0030213 0.0082178
CYC2Ba-CYC2A   -0.5104260 -1.73793277 0.7170808 0.8115940
CYC2Bb-CYC2A    0.3654424 -0.73247308 1.4633578 0.9160458
CYC3A-CYC2A    -0.2175709 -1.31548638 0.8803445 0.9908727
CYC3B-CYC2A     0.9798406 -0.24766622 2.2073474 0.1842674
CYC2Bb-CYC2Ba   0.8758683 -0.35163845 2.1033751 0.2899105
CYC3A-CYC2Ba    0.2928551 -0.93465174 1.5203618 0.9790010
CYC3B-CYC2Ba    1.4902665  0.14560023 2.8349329 0.0223434
CYC3A-CYC2Bb   -0.5830133 -1.68092874 0.5149022 0.6092075
CYC3B-CYC2Bb    0.6143982 -0.61310859 1.8419050 0.6665353
CYC3B-CYC3A     1.1974115 -0.03009529 2.4249183 0.0594548
```

##
##

ANOVA of relative expression levels for six different CYC-like genes comparing internal bud, external bud, and leaf tissue.

```
               Df Sum Sq Mean Sq F value Pr(>F)
CYC1.bl$flower  2  14.89   7.444   6.832 0.0062 **
Residuals      18  19.61   1.090

Signif. codes:  0 ‘***’ 0.001 ‘**’ 0.01 ‘*’ 0.05 ‘.’ 0.1 ‘ ’ 1

  Tukey multiple comparisons of means
    95% family-wise confidence level

CYC1.bl$flower
                        diff        lwr        upr     p adj
internal-external -2.0234122 -3.4274504 -0.6193739 0.0046586
leaf-external     -0.6226772 -2.0267154  0.7813610 0.5075446
leaf-internal      1.4007350 -0.1373119  2.9387818 0.0778516
```

__

```
                Df Sum Sq Mean Sq F value  Pr(>F)
CYC2A.bl$flower  2  9.862   4.931   8.179 0.00297 **
Residuals       18 10.851   0.603

Signif. codes:  0 ‘***’ 0.001 ‘**’ 0.01 ‘*’ 0.05 ‘.’ 0.1 ‘ ’ 1

  Tukey multiple comparisons of means
    95% family-wise confidence level

CYC2A.bl$flower
                         diff       lwr        upr     p adj
internal-external  0.02618554 -1.018205  1.0705764 0.9977454
leaf-external     -1.50624742 -2.550638 -0.4618565 0.0046305
leaf-internal     -1.53243296 -2.676506 -0.3883600 0.0081876
```

__

```
                  Df Sum Sq Mean Sq F value  Pr(>F)
CYC2Ba.bl$flower   2  33.93  16.967   17.87 5.3e-05 ***
Residuals         18  17.09   0.949

Signif. codes:  0 ‘***’ 0.001 ‘**’ 0.01 ‘*’ 0.05 ‘.’ 0.1 ‘ ’ 1

  Tukey multiple comparisons of means
    95% family-wise confidence level

CYC2Ba.bl$flower
                        diff       lwr        upr     p adj
internal-external -0.7478289 -2.058453  0.5627955 0.3346110
leaf-external     -3.0282832 -4.338907 -1.7176589 0.0000397
```

```
leaf-internal     -2.2804543 -3.716171 -0.8447373 0.0020467
```

```
                 Df Sum Sq Mean Sq F value  Pr(>F)
CYC2Bb.bl$flower  2  62.99  31.493   28.83 9.5e-07 ***
Residuals        21  22.94   1.092

Signif. codes:  0 ‘***’ 0.001 ‘**’ 0.01 ‘*’ 0.05 ‘.’ 0.1 ‘ ’ 1

  Tukey multiple comparisons of means
    95% family-wise confidence level

CYC2Bb.bl$flower
                        diff        lwr       upr     p adj
internal-external  0.2314342 -0.9302119  1.393080 0.8710629
leaf-external     -4.7884903 -6.4889660 -3.088015 0.0000015
leaf-internal     -5.0199244 -6.7761682 -3.263681 0.0000012
```

```
                Df Sum Sq Mean Sq F value Pr(>F)
CYC3A.bl$flower  2  2.509  1.2544   1.831  0.194
Residuals       15 10.277  0.6851

  Tukey multiple comparisons of means
    95% family-wise confidence level

CYC3A.bl$flower
                         diff        lwr      upr     p adj
internal-external -0.02349016 -1.1566188 1.109638 0.9984029
leaf-external      0.99197063 -0.4413363 2.425278 0.2039449
leaf-internal      1.01546079 -0.5047908 2.535712 0.2250263
```

```
                 Df Sum Sq Mean Sq F value Pr(>F)
CYC3B.bl$flower  2   6.43   3.213   0.641  0.538
Residuals        18  90.16   5.009

  Tukey multiple comparisons of means
    95% family-wise confidence level

CYC3Bb.bl$flower
                        diff       lwr      upr     p adj
internal-external -0.5196680 -3.530077 2.490741 0.8991258
leaf-external     -1.3358482 -4.346258 1.674561 0.5071675
leaf-internal     -0.8161802 -4.113919 2.481558 0.8048241

##########################################################################################
##########################################################################################
```

ANOVA comparisons of CYC-like genes by petal type in internal and external florets.

```
             Df Sum Sq Mean Sq F value  Pr(>F)
CYC1.i$petal  3 12.617   4.206   7.124 0.00527 **
Residuals    12  7.085   0.590

Signif. codes:  0 ‘***’ 0.001 ‘**’ 0.01 ‘*’ 0.05 ‘.’ 0.1 ‘ ’ 1

  Tukey multiple comparisons of means
    95% family-wise confidence level

CYC1.i$petal
            diff        lwr       upr     p adj
LL-D   2.3584327  0.7453801 3.9714852 0.0045903
RL-D   2.0375633  0.2952674 3.7798591 0.0207894
```

```
V-D    1.1101902 -0.7524024 2.9727828 0.3332235
RL-LL -0.3208694 -1.7933782 1.1516393 0.9146006
V-LL  -1.2482424 -2.8612950 0.3648101 0.1531578
V-RL  -0.9273730 -2.6696689 0.8149228 0.4246444

——
             Df Sum Sq Mean Sq F value   Pr(>F)    
CYC1.e$petal  3  19.43   6.477   9.623 0.000445 ***
Residuals    19  12.79   0.673                     

Signif. codes:  0 ‘***’ 0.001 ‘**’ 0.01 ‘*’ 0.05 ‘.’ 0.1 ‘ ’ 1

  Tukey multiple comparisons of means
    95% family-wise confidence level

CYC1.e$petal
            diff        lwr         upr     p adj
LL-D   0.4788492 -0.9179708  1.87566930 0.7710926
RL-D   0.9062899 -0.4905301  2.30311000 0.2930337
V-D   -1.4798696 -2.8766897 -0.08304952 0.0355388
RL-LL  0.4274407 -0.9043750  1.75925638 0.8036812
V-LL  -1.9587188 -3.2905345 -0.62690315 0.0028938
V-RL  -2.3861595 -3.7179752 -1.05434385 0.0003916

——
              Df Sum Sq Mean Sq F value   Pr(>F)    
CYC2A.i$petal  3  19.49   6.497   8.796 0.000568 ***
Residuals     21  15.51   0.739                     

Signif. codes:  0 ‘***’ 0.001 ‘**’ 0.01 ‘*’ 0.05 ‘.’ 0.1 ‘ ’ 1

  Tukey multiple comparisons of means
    95% family-wise confidence level

CYC2A.i$petal
            diff       lwr        upr     p adj
LL-D  -1.3113960 -2.750951  0.1281593 0.0824898
RL-D  -1.9437002 -3.490031 -0.3973698 0.0105091
V-D   -2.7396642 -4.285995 -1.1933338 0.0003755
RL-LL -0.6323043 -1.894878  0.6302692 0.5155622
V-LL  -1.4282682 -2.690842 -0.1656948 0.0229348
V-RL  -0.7959640 -2.179044  0.5871160 0.3978429

——
              Df Sum Sq Mean Sq F value   Pr(>F)    
CYC2A.e$petal  3  77.25  25.750   38.27 7.48e-11 ***
Residuals     33  22.20   0.673                     

Signif. codes:  0 ‘***’ 0.001 ‘**’ 0.01 ‘*’ 0.05 ‘.’ 0.1 ‘ ’ 1

  Tukey multiple comparisons of means
    95% family-wise confidence level

CYC2A.e$petal
             diff        lwr        upr     p adj
LL-D  -1.54551191 -2.6006861 -0.4903377 0.0020275
RL-D  -1.48746083 -2.6055508 -0.3693709 0.0054408
V-D   -4.23193858 -5.3500285 -3.1138487 0.0000000
RL-LL  0.05805108 -0.9202776  1.0363798 0.9984973
V-LL  -2.68642667 -3.6647554 -1.7080980 0.0000001
V-RL  -2.74447774 -3.7903551 -1.6986004 0.0000002

——
                Df Sum Sq Mean Sq F value Pr(>F)  
CYC2Ba.i$petal  3  8.513  2.8378   3.646 0.0354 *
Residuals      16 12.452  0.7782                 

Signif. codes:  0 ‘***’ 0.001 ‘**’ 0.01 ‘*’ 0.05 ‘.’ 0.1 ‘ ’ 1
```

```
  Tukey multiple comparisons of means
    95% family-wise confidence level

CYC2Ba.i$petal
            diff        lwr        upr     p adj
LL-D   1.5769099 -0.2077552 3.36157497 0.0932054
RL-D   1.0460746 -0.6626121 2.75476132 0.3311728
V-D   -0.1293874 -2.1901411 1.93136637 0.9978532
RL-LL -0.5308353 -1.8938958 0.83222517 0.6863036
V-LL  -1.7062973 -3.4909623 0.07836783 0.0634862
V-RL  -1.1754619 -2.8841487 0.53322477 0.2404640

__
                Df Sum Sq Mean Sq F value   Pr(>F)    
CYC2Ba.e$petal  3  52.41   17.47   46.03 3.78e-11 ***
Residuals       29  11.01    0.38                     

Signif. codes:  0 ‘***’ 0.001 ‘**’ 0.01 ‘*’ 0.05 ‘.’ 0.1 ‘ ’ 1

  Tukey multiple comparisons of means
    95% family-wise confidence level

CYC2Ba.e$petal
             diff        lwr        upr     p adj
LL-D   1.76848803  0.8838818  2.6530943 0.0000417
RL-D   1.74326519  0.9040540  2.5824764 0.0000232
V-D   -1.41979653 -2.3888341 -0.4507589 0.0021923
RL-LL -0.02522283 -0.7653375  0.7148919 0.9997062
V-LL  -3.18828456 -4.0728908 -2.3036783 0.0000000
V-RL  -3.16306173 -4.0022729 -2.3238505 0.0000000

__
               Df Sum Sq Mean Sq F value   Pr(>F)    
CYC2Bb.i$petal  3 20.665   6.888   26.74 4.98e-07 ***
Residuals      19  4.894   0.258                     

Signif. codes:  0 ‘***’ 0.001 ‘**’ 0.01 ‘*’ 0.05 ‘.’ 0.1 ‘ ’ 1

  Tukey multiple comparisons of means
    95% family-wise confidence level

CYC2Bb.i$petal
            diff        lwr        upr     p adj
LL-D   0.0589175 -0.7118271  0.8296621 0.9963679
RL-D  -0.2212295 -1.1874089  0.7449500 0.9163896
V-D   -2.1683954 -2.9391400 -1.3976508 0.0000011
RL-LL -0.2801470 -1.2892885  0.7289945 0.8622781
V-LL  -2.2273129 -3.0512735 -1.4033523 0.0000020
V-RL  -1.9471659 -2.9563074 -0.9380244 0.0001682

__
               Df Sum Sq Mean Sq F value   Pr(>F)    
CYC2Bb.e$petal  3  52.89  17.630   43.06 8.32e-11 ***
Residuals      29  11.87   0.409                     

Signif. codes:  0 ‘***’ 0.001 ‘**’ 0.01 ‘*’ 0.05 ‘.’ 0.1 ‘ ’ 1

  Tukey multiple comparisons of means
    95% family-wise confidence level

CYC2Bb.e$petal
             diff        lwr        upr     p adj
LL-D   0.18688718 -0.6978755  1.0716498 0.9386155
RL-D   0.05923257 -0.7836463  0.9021115 0.9974534
V-D   -2.77496944 -3.5585284 -1.9914105 0.0000000
RL-LL -0.12765461 -1.0975420  0.8422328 0.9838874
V-LL  -2.96185662 -3.8806610 -2.0430523 0.0000000
```

```
V-RL  -2.83420201 -3.7127470 -1.9556570 0.0000000

---
              Df Sum Sq Mean Sq F value Pr(>F)
CYC3A.i$petal  3  5.939  1.9797   3.597 0.0433 *
Residuals     13  7.154  0.5503

Signif. codes:  0 '***' 0.001 '**' 0.01 '*' 0.05 '.' 0.1 ' ' 1

  Tukey multiple comparisons of means
    95% family-wise confidence level

CYC3A.i$petal
             diff        lwr         upr     p adj
LL-D  -1.58557433 -3.1252247 -0.04592396 0.0427527
RL-D   0.08606563 -1.4535847  1.62571601 0.9983448
V-D   -0.50657487 -1.8250527  0.81190294 0.6797275
RL-LL  1.67163997 -0.1061952  3.44947509 0.0681726
V-LL   1.07899946 -0.5111446  2.66914353 0.2404103
V-RL  -0.59264050 -2.1827846  0.99750357 0.6992393

---
              Df Sum Sq Mean Sq F value Pr(>F)
CYC3A.e$petal  3  1.168  0.3892    0.93  0.443
Residuals     22  9.211  0.4187

  Tukey multiple comparisons of means
    95% family-wise confidence level

CYC3A.e$petal
              diff        lwr       upr     p adj
LL-D  -0.207221835 -1.1775940 0.7631503 0.9331296
RL-D  -0.663542964 -1.8799688 0.5528829 0.4460540
V-D   -0.003898399 -0.8769766 0.8691798 0.9999993
RL-LL -0.456321129 -1.7268365 0.8141942 0.7524567
V-LL   0.203323436 -0.7436628 1.1503097 0.9321423
V-RL   0.659644565 -0.5382088 1.8574980 0.4379336

---
               Df Sum Sq Mean Sq F value Pr(>F)
CYC3B.i$petal  3  1.711  0.5703     2.2  0.128
Residuals      16  4.147  0.2592

  Tukey multiple comparisons of means
    95% family-wise confidence level

CYC3B.i$petal
             diff        lwr       upr     p adj
LL-D  -0.47711629 -1.5070705 0.5528379 0.5609739
RL-D  -0.37978686 -1.3658930 0.6063192 0.6934979
V-D   -1.04434175 -2.2336304 0.1449469 0.0958863
RL-LL  0.09732943 -0.6893111 0.8839700 0.9842306
V-LL  -0.56722546 -1.5971797 0.4627288 0.4190799
V-RL  -0.66455489 -1.6506610 0.3215512 0.2557266

---
               Df Sum Sq Mean Sq F value   Pr(>F)
CYC3B.e$petal  3  17.65   5.882   13.47 2.77e-05 ***
Residuals      23  10.04   0.437

Signif. codes:  0 '***' 0.001 '**' 0.01 '*' 0.05 '.' 0.1 ' ' 1

  Tukey multiple comparisons of means
    95% family-wise confidence level

CYC3B.e$petal
            diff       lwr        upr     p adj
LL-D  -0.4455396 -1.409283  0.5182036 0.5847382
```

RL-D	-0.8808745	-1.936602	0.1748532	0.1252146
V-D	-2.2544612	-3.310189	-1.1987334	0.0000283
RL-LL	-0.4353349	-1.399078	0.5284082	0.6025744
V-LL	-1.8089216	-2.772665	-0.8451784	0.0001593
V-RL	-1.3735866	-2.429314	-0.3178589	0.0076513
